# Acute and Chronic Sarcoid Arthropathies: Characteristics and Treatments From a Retrospective Nationwide French Study

**DOI:** 10.3389/fmed.2020.565420

**Published:** 2020-12-10

**Authors:** Carlotta Cacciatore, Pierre Belnou, Sara Thietart, Carole Desthieux, Mathilde Versini, Noemie Abisror, Sébastien Ottaviani, Gregoire Cormier, Alban Deroux, Azeddine Dellal, Nicolas Belhomme, Nathalie Saidenberg Kermanac'H, Philippe Khafagy, Martin Michaud, Sylvain Lanot, Fabrice Carrat, Olivier Fain, Arsène Mékinian

**Affiliations:** ^1^Sorbonne Université, Service de médecine interne, Hôpital Saint-Antoine, DHU I2B: Inflammation, Immunopathologie, Biothérapie, APHP, Paris, France; ^2^Sorbonne Université, Service de santé publique, Hôpital Saint-Antoine, APHP, Paris, France; ^3^Service de Médecine interne, Centre Hospitalier Universitaire de Nice, Nice, France; ^4^Service de rhumatologie, Hôpital Bichat, APHP, Paris, France; ^5^Service de rhumatologie, CHD Vendée, La Roche-sur-Yon, France; ^6^Service de médecine interne, CHU Grenoble, La Tronche, France; ^7^Service de rhumatologie, Hôpital Montfermeil, Montfermeil, France; ^8^Service de médecine interne et immunologie clinique, CHU Rennes, Rennes, France; ^9^Service de rhumatologie, Groupe hospitalier Avicenne-Jean Verdier-René Muret, APHP, Bobigny, France; ^10^Sorbonne Paris Cité, Université Paris 13, INSERM U1125, Bobigny, France; ^11^Service de radiologie, Hôpital Montfermeil, Montfermeil, France; ^12^Service de médecine interne, Hôpital Joseph Ducuing, Toulouse, France; ^13^Service de rhumatologie, C.H intercommunal Alençon-Mamers, Alençon, France; ^14^Sorbonne Université, INSERM, Institut Pierre Louis d'Epidémiologie et de Santé Publique, Paris, France

**Keywords:** sarcoid arthropathy, outcome, methotrexate, infliximab, sarcoidosis

## Abstract

**Introduction:** We aimed to analyze patients with acute and chronic joint involvements in sarcoidosis.

**Methods:** This is a retrospective multicenter analysis of patients with proven sarcoidosis, as defined by clinical, radiological, and histological criteria, with at least one clinical and/or ultrasonographic synovitis.

**Results:** Thirty-nine patients with sarcoid arthropathy were included, and among them 19 had acute sarcoidosis (Lofgren's syndrome). Joint involvement and DAS44-CRP were not significantly different in acute and chronic sarcoid arthropathies. Acute forms were more frequent than chronic sarcoid arthropathy in Caucasians, without any difference of sex or age between these 2 forms. Joint involvement was frequently more symmetrical in acute than chronic forms (100 vs. 70%; *p* < 0.05), with a more frequent involvement in wrists and ankles in acute forms, whereas the tender and swollen joint counts and the DAS44-CRP were similar between the 2 groups. Skin lesions were significantly more frequent in patients with acute forms [17 (89%) vs. 5 (25%); *p* < 0.05] and were erythema nodosum in all patients with Löfgren's syndrome and sarcoid skin lesions in those with chronic sarcoidosis. Among 20 patients with chronic sarcoidosis, treatment was used in 17 (85%) cases, and consisted in NSAIDs alone (*n* = 5; 25%), steroids alone (*n* = 5; 25%), hydroxychloroquine (*n* = 2; 20%), methotrexate (*n* = 3; 15%), and TNF inhibitors (*n* = 2; 10%). A complete/partial joint response was noted in 14 (70%) cases with a DAS44-CRP reduction of 2.07 [1.85–2.44] (from 3.13 [2.76–3.42] to 1.06 [0.9–1.17]; *p* < 0.05).

**Conclusion:** Sarcoid arthropathies have different clinical phenotypes in acute and chronic forms and various treatment regimens such as hydroxychloroquine and methotrexate could be used in chronic forms.

## Introduction

Sarcoidosis is a heterogeneous systemic granulomatous disease affecting mostly lung and lymphatic nodes. Non-caseating granulomas are the characteristic histopathological feature. Joint involvement, also known as sarcoid arthropathy, is observed in 6–35% of patients, and asymptomatic bone involvement in 3–13% of patients. Acute-onset arthritis is generally characterized by symmetric arthritis, and in Lofgren's syndrome is associated with bilateral hilar adenopathies and erythema nodosum, with remission usually occurring within 6–10 weeks (1–5). Chronic sarcoid arthropathy is characterized by persistent oligo or poly-arthritis in 20% of patients, with 40% of arthralgia (1, 2). Few studies have described the prevalence and features of sarcoid arthropathies, and association with other organ involvements (2, 4, 6, 7). Non-steroidal anti-inflammatory drugs (NSAIDs) are effective in acute sarcoidosis, but little is known about the efficacy of other therapies in steroid-dependent and refractory forms of sarcoid arthropathy. We aimed to describe clinical characteristics of sarcoid arthropathies and describe the treatment.

## Patients and Methods

### Study Scheme

The study was observational with a retrospective analysis. A call for observation was sent to members of the “French Inflammatory Joint Disease Working Group” (Club Rhumatismes et Inflammation) and the National French Society of Internal Medicine (SNFMI) from July 2017 to November 2018. Physicians were asked to fill in a defined table. The study complied with the recommendations of the Declaration of Helsinki, and being observational and retrospective, ethics committee approval was not necessary according to the French local law. Ethical review and approval was not required for the study on human participants in accordance with the local legislation and institutional requirements. Written informed consent from the patients was not required to participate in this study in accordance with the national legislation and the institutional requirements.

### Patients

Patients were included if they were aged of 18 years or more, had histologically-proven sarcoidosis (except for typical acute forms of Löfgren syndrome) and at least one episode of arthritis, defined as the presence of clinical and/or ultrasonographic synovitis (8–11). The definition of ultrasonographic synovitis was used as usually done in patients with rheumatoid arthritis or other inflammatory arthritis. Exclusion criteria were: patients with other granulomatous diseases, associated rheumatoid arthritis, systemic lupus erythematosus, mixed connective tissue disease, microcrystalline arthritis or infectious arthritis (3). All patients' medical records were reviewed by 2 investigators (Carlotta Cacciatore and Arsène Mekinian).

### Data Collection

Clinical data was collected as follows: age, ethnicity, sex, date of diagnosis, disease duration, and presence of skin, lung, heart, ocular, and neurological involvements. Articular assessment, which was collected at diagnosis and during follow-up, included: tender joint count, swollen joint count, localization of joint involvement and DAS44-CRP. Synovitis was diagnosed by a clinical examination and/or joint ultrasonography: among 39 included patients with clinical synovitis, 31 have undergone joint ultrasonography and have all ultrasound-confirmed synovitis. Synovitis was defined as synovial hypertrophy ≥2, or synovial hypertrophy ≥1+PD signal ≥1, according to the OMERACT definitions (12). Joint activity was quantified using DAS28-CRP at diagnosis and during the follow up. The use of non-steroidal anti-inflammatory drugs (NSAIDs), steroids and/or synthetic disease-modifying antirheumatic drugs (DMARDs), hydroxychloroquine and TNF antagonists was collected. Laboratory data included serum hemoglobin, platelet, neutrophil and lymphocyte counts, calcium, creatinine, alanine aminotransferase, aspartate aminotransferase, C-reactive protein (CRP), erythrocyte sedimentation rates (ESR), gamma-globulin, angiotensin converting enzyme (ACE), rheumatoid factor, antinuclear and anti-citrullinated protein antibodies (ACPA). Sarcoidosis was considered as acute if remission was completely reached within 12 weeks. Löfgren's syndrome was defined in patients with acute sarcoidosis in the presence of arthritis, bilateral hilar adenopathy and erythema nodosum, and fever (6, 13). All remaining cases were defined as chronic sarcoid arthropathy. Efficacy of each drug was compared to each other, and all treatment lines were analyzed. Complete articular response to treatment was defined as total disappearance of arthralgia and synovitis, with a DAS44-CRP <1.6 (14, 15). Partial response was defined as an improvement of at least 50% of swollen joint count (i.e., number of synovitis). Non-response was defined as all remaining cases. For each line of treatment, the reasons of interruption has been codified among inefficacy, adverse events and remission.

### Statistical Analysis

Data is expressed as medians with ranges and numbers with frequencies. Student *t-*test or Wilcoxon-Mann Whitney test were used to compare quantitative data and Chi-square or Fisher's exact tests for qualitative variables. All analyses were done using R software (R Foundation, Vienna, Austria version 3.0.2) and a *p* < 0.05 was considered as significant.

## Results

Thirty-nine patients (64% women) were included with a median age of 41 years [25–75] (Table 1). Among 39 patients with clinical arthritis, 31 (82%) had done ultrasound echography and all these 31 cases had ultrasound-confirmed synovitis. The pattern of arthritis was mostly symmetric polyarthritis affecting ankles in 33 (85%) cases, wrists in 18 (46%) cases and metacarpo-phalangeal joints in 12 (31%) cases. None reported spine involvement or dactylitis. Median tender and swollen joint count at diagnosis was 6 [1–28] and 2 [1–6], respectively. C-reactive protein levels were at 22 mg/l [1–271] and ACE at 65 U/L [21.2–300]. Median DAS44-CRP at diagnosis was 3.4 [2.3–5.9]. No patients had positive rheumatoid factor or ACPA, nor radiological structural damage. Acute forms were diagnosed in 19 (49%) patients and among them 17 (89%) have Löfgren syndrome. The remaining 20 (51%) patients have chronic sarcoid arthropathy. Acute forms were more frequent than chronic sarcoid arthropathies in caucasians, without any difference of sex or age between these 2 forms. Joint involvement was frequently more symmetrical in acute than chronic forms (100 vs. 70%; *p* < 0.05), with a more frequent involvement in wrists and ankles in acute forms, whereas the tender and swollen joint counts and the DAS44-CRP were similar between the 2 groups (Table 1). Skin lesions were significantly more frequent in patients with acute forms [17 (89%) vs. 5 (25%); *p* < 0.05] and were erythema nodosum in all patients with Löfgren's syndrome and sarcoid skin lesions in those with chronic sarcoidosis. Ocular involvement was present only in patients with chronic sarcoidosis (*n* = 4, 20%): one chorioretinitis and anterior uveitis (*n* = 3). There were no significant differences in calcium levels, ACE, gammaglobulin, and lymphocyte levels at diagnosis between patients with acute and chronic sarcoid arthropathies (data not shown).

**Table 1:** Characteristics of patients with joint involvement and acute/chronic sarcoidosis.

**Characteristics**	**All patients** ***N* = 39**	**Acute sarcoidosis** ***N* = 19**	**Chronic sarcoidosis** ***N* = 20**
Age (years), medians [ranges]	38 [23–70]	34.5 [23–70]	42 [25–65]
Female sex (*n*;%)	25 (64)	13 (68)	12 (60)
Caucasians (*n*;%)	19 (48.7)	12 (63)	7 (35)^*^
Extra-articular involvement			
Skin involvement (*n*;%)	22 (56)	17 (89)	5 (25)^*^
Ocular involvement (*n*;%)	4 (10)	0	4 (20)^*^
Hilar and mediastinal adenopathies (*n*;%) Lung involvement (*n*;%)	19 (49) 32 (82)	8 (42) 16 (84)	11 (55)^*^ 16 (80)
Heart involvement (*n*;%) CNS involvement (*n*;%)	2 (5) 1 (2.5)	0 0	2 (10) 1 (5)
Joint involvement			
Symmetrical (*n*;%) Wrist (*n*;%) Ankles (*n*;%) Metacarpo-phalangeal (*n*;%) Knees (*n*;%)	33 (85) 18 (46) 32 (82) 12 (31) 16 (41)	19 (100) 5 (26) 17 (89) 4 (21) 6 (32)	14 (70)^*^ 10 (50)^*^ 13 (65)^*^ 6 (30) 10 (50)
Tender joints medians [ranges]	6 [1–28]	4 [2–28]	6 [1–12]
Swollen joints medians [ranges]	2 [1–6]	2 [1–4]	2 [1–6]
DAS 44-CRP medians [ranges]	3.4 [2.3–5.9]	3.7 [2.3–5.9]	3.4 [2.3–3.5]
Laboratory data			
Lymphocytes (G/l) medians [ranges]	1.410 [0.66–3.35]	1.8 [0.66–2.50]	1.11 [0.71–3.35]
Gammaglobulins (g/l) medians [ranges]	11.6 [7.5–24.7]	11.4 [7.5–13.3]	12.3 [7.8–24.7]
Calcium levels (mg/l) medians [ranges]	2.34 [2.16–2.57]	2.3 [2.16–2.53]	2.36 [2.26–2.57]
ACE (UI/l) medians [ranges]	65 [21.2–300]	55 [21.20–111]	76.5 [36–300]
First line treatments	34 (87)	17 (89)	17 (85)
NSAIDs alone (*n*;%)	15 (38)	10 (53)	5 (25)^*^
Steroids alone (*n*;%)	9 (23)	4 (21)	5 (25)
Hydroxychloroquine (*n*;%) With NSAIDs With steroids	4 (10) 3 (7.5) 0	2 (10) 2 (10) 0	2 (10) 1 (5) 0
Methotrexate (*n*;%) With NSAIDs With steroids	4 (10) 1 (2.5) 2 (5)	1 (5) 1 (5) 0	3 (15) 0 2 (10)
TNF inhibitors (*n*;%) With steroids	2 (5)	0 0	5 (25) 5 (10)
Follow-up (months) medians [ranges]	18 [3–264]	20.5 [3–57]	62.5 [3–264]^*^

*Values are medians with interquartiles and numbers with frequencies*.

* *p < 0.05*.

*ACPA, anti Citrullinated Peptide antibodies; ANA, anti-nuclear antibodies; CRP, C-reactive protein; ESR, erythrocyte-sedimentation rate; NSAIDs, non-steroidal anti-inflammatories; RF, rheumatoid factor*.

During the median follow-up of 18 [3–264] months, 34 patients (87%) received at least one therapy among NSAIDs, steroids, methotrexate, hydroxychloroquine or TNF inhibitors. Among the 19 patients with acute sarcoidosis, treatment was used in 17 (89%) cases, and consisted of NSAIDs alone (*n* = 10; 53%), steroids alone (*n* = 4; 21%), hydroxychloroquine (*n* = 2; 10%), and methotrexate (*n* = 1; 5%) (Table 1). A complete/partial joint response was noted in 15 (79%) cases with a DAS44-CRP reduction of 2.45 [1.66–2.66] (from 3.37 [2.62–3.48] to 0.92 [0.89–1.58]; *p* < 0.05).

Among 20 patients with chronic sarcoidosis, a first-line treatment was used in 17 (85%) cases, and consisted of NSAIDs alone (*n* = 5; 25%), steroids alone (*n* = 5; 25%), hydroxychloroquine (*n* = 2; 20%), methotrexate (*n* = 3; 15%), and TNF inhibitors (*n* = 5; 25%) (Table 1). A complete/partial joint response was noted in 14 (70%) cases with a DAS44-CRP reduction of DAS44-CRP reduction of 2.07 [1.85–2.44] (from 3.13 [2.76–3.42] to 1.06 [0.9–1.17]; *p* < 0.05). A second-line therapy for chronic sarcoidosis was used in 8 cases: methotrexate (*n* = 6), steroids alone and hydroxychloroquine (*n* = 1 each) with a joint response in 6 cases. A third-line therapy for sarcoid chronic arthropathy was used in 5 cases and among them TNF inhibitors in 5 cases with joint responses in all cases.

**Figure 1:**
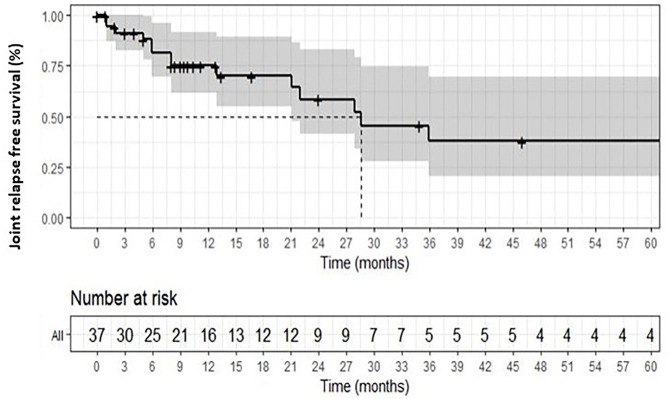
Overall relapse-free survival in sarcoid arthropathy.

NSAIDs was the most frequent first-line option in acute sarcoidosis [10 (53%) vs. 5 (25%); *p* < 0.05], but other first-line therapy frequencies were not significantly different between the 2 groups. The median time of treatment was at 2.5 months [0.6–7]. The joint relapse-free survival was at 26.6 months in the overall group (Figure 1).

## Discussion

This study reports clinical and laboratory features of acute and chronic sarcoid arthropathies, and shows some significant differences in joint and extra-articular involvements. The acute forms have a more symmetrical joint distribution with predominant skin involvement, whereas chronic sarcoid arthropathies have more frequent ocular involvements (16).

Joint involvement in sarcoidosis ranges from 6 to 35% (2, 11, 13–15, 17–19). Clinical features of acute and chronic sarcoid arthropathies have rarely been analyzed (20, 21). The chronic form is usually associated with parenchymal lung or other organ involvements and is relatively rare (up to 7%) (4, 18, 22, 23). In our study, swollen joint count and DAS44 CRP were comparable in both sarcoid arthropathies, but acute forms were frequently more symmetrical and affected ankles (4). DAS-28 score is not validated in the setup of sarcoidosis, and in particular does not include the ankle involvement which could be frequent in acute forms, but has already previously been used in other inflammatory arthritis and sarcoid arthritis (24, 25). Consistently with previous studies, skin involvement (mostly erythema nodosum) was particularly frequent in acute sarcoid arthropathy, whereas ocular involvement occurred just in chronic subsets (26). The presence and features of hilar and lung involvements were not discriminatory between acute and chronic sarcoid forms.

Management of sarcoid arthropathy is poorly studied (11, 27): most data involves acute forms, with chronic forms only described in small case-series. In this study, we compared the efficacy of various treatment regimens on sarcoid arthropathy with clinical synovitis. Using pooled lines of various therapies, we showed no significant differences in joint responses using hydroxychloroquine, methotrexate and TNF antagonists. Previous studies confirmed the efficacy of NSAIDs and steroids for acute sarcoid arthropathy (13, 18, 28), as we reported in the present study. For chronic sarcoid arthropathy, small case-series showed the benefit of methotrexate (29, 30), whereas in a retrospective study among 10 patients treated with TNF antagonists, despite initial rapid efficacy, no clinical amelioration was noted after 1 year of treatment (24). Judson et al. conducted the only trial evaluating sarcoidosis joint manifestations under infliximab. No satisfactory effect on joint disease was observed at 24 and 48 weeks, even though significant attenuation of involvement for all combined organs was observed at 28 weeks (22). TNF- antagonists could also have a steroid-sparing effect, in particular in chronic sarcoidosis (22, 24, 25).

Our study has several biases that limit definitive conclusions. Firstly, it is a retrospective study with small sample sizes, and the small number of patients treated by TNF antagonists does not allow definitive conclusions about efficacy of this therapy in joint involvement. We assessed disease activity with DAS44-CRP score usually used for rheumatoid arthritis, as some other previous studies (24), because no validated score exists to evaluate joint activity in sarcoidosis.

## Conclusion

Sarcoid arthropathies have different clinical phenotypes in acute and chronic forms and various treatment regimens such as hydroxychloroquine and methotrexate could be used in chronic forms.

## Data Availability Statement

The raw data supporting the conclusions of this article will be made available by the authors, without undue reservation.

## Ethics Statement

Ethical review and approval was not required for the study on human participants in accordance with the local legislation and institutional requirements. Written informed consent from the patients was not required to participate in this study in accordance with the national legislation and the institutional requirements.

## Author Contributions

All authors listed have made a substantial, direct and intellectual contribution to the work, and approved it for publication.

## Conflict of Interest

The author declares that the research was conducted in the absence of any commercial or financial relationships that could be construed as a potential conflict of interest.
