# Supplementary material for: Acute and Chronic Sarcoid Arthropathies: Characteristics and Treatments From a Retrospective Nationwide French Study
Source: Front Med (Lausanne). 2020 Dec 10;7:565420. doi: 10.3389/fmed.2020.565420 (PMC7758528; doi:10.3389/fmed.2020.565420)
Supplement: Supplementary file 1 [file Table_1.pdf]

Characteristics	All lines of treatment N = 25	Steroids N =6	Hydroxychloroquine N = 4	Methotrexate N = 10	Infliximab N = 5
**Tender joints before/at the end of each line of treatment**	6 [1-8]<br>0 [0-12]*	6 [1-8]<br>0 [0-2]*	1 [0-4]<br>0 [0-1]	5 [2-12]<br>3.5 [0-8]*	6 [0-8]<br>0 [0-12]
**Swollen joints before / at the end of each line of treatment**	2 [1-6]<br>0 [0-3]*	1.5 [1-6]<br>0 [0-1]*	0 [0-2]<br>0 [0-1]	2 [0-6]<br>0 [0-3]*	2 [0-6]<br>0 [0-2]
**DAS 44 -CRP before/at the end of each line of treatment**	3.3 [2.76-3.62]<br>1.06 [0.9-1.17]*	3.21 [2.76-3.78]<br>1 [0.9-1.13]*	2.46[2.17-2.75]<br>1.15[1.07-1.17]	3.33 [2.8-3.9]<br>1.07 [0.9-1.4]*	4.1 [3.8-4.2]<br>1.6 [1.6-1.8]**
**Prednisone (mg/day) before/at the end of each line of treatment**	10 [5-80]<br>1.5 [0-50]*	40 [5-70]<br>0 [0-50]*	2.5 [0-5]<br>1 [0-5]	10 [5-80]<br>2.5 [0-10]*	7.5 [5-10]<br>0 [0-5]*

*p<0.05 (no statistical analysis for hydroxychloroquine subgroup because of small number of patients)
